# Solid-State Form Design in Pharmaceutical Drug Product Development

**DOI:** 10.1063/4.0001081

**Published:** 2025-10-27

**Authors:** Rajni Miglani Bhardwaj

**Affiliations:** Pfizer, Groton, CT, USA

## Abstract

Pharmaceutical molecules have the miraculous property to exist in multiple solid-state forms i.e. polymorphs, hydrates, salts, co-crystals. These solid-state forms may exhibit different physical, chemical, kinetic, surface, and mechanical properties, which can lead to differences in bioavailability, manufacturability, and stability of drug product. Therefore, it is very crucial to select the solid form with optimum properties for a given drug product and which can be produced reliably. Over the past decade, pharmaceutical industry has witnessed an ever-increasing utilization of computational tools i.e. crystal structure prediction, solid form informatics etc. to complement experimental solid form selection process. In this presentation, we will be discussing how experimental and computational approaches are used in conjunction for solid form design and selection. Case studies will be presented where combined application of experimental and computational approaches were used in conjunction to underpin the link between the structural features and derived properties and to highlight how computational tools provide an opportunity to gain fundamental understanding at molecular and crystal structure level which can help with designing of robust drug product.

